# 造血干细胞移植后长期合并症管理中国专家共识（2023年版）

**DOI:** 10.3760/cma.j.issn.0253-2727.2023.09.003

**Published:** 2023-09

**Authors:** 中华医学会血液学分会干细胞应用学组

近年来，随着造血干细胞移植（以下简称移植）技术的进步，移植后长期生存人数得以持续增加。移植后生存2年以上且无原发病复发的患者中约80％获长期生存，但移植后长期生存者仍面临心血管系统疾病、呼吸系统疾病、内分泌疾病及继发二次肿瘤等长期合并症的风险[1]–[3]。长期合并症的发生受多种移植治疗因素的影响，包括预处理用药、移植物抗宿主病（GVHD）防治用药等，也与患者年龄、性别、移植前合并症等相关。移植后长期合并症的出现直接影响到长期生存者的生活质量及非复发死亡率。因此，对移植后长期生存者进行终身随访，筛查和管理移植后长期并发症是极为必要的。本共识重点介绍不同系统长期合并症的发病率、危险因素、临床表现及随访建议。

一、心血管疾病

（一）心血管疾病发病率、高危因素

移植后最常见的心血管疾病为冠状动脉粥硬化性心脏病（冠心病）和心功能不全。移植后长期生存者心血管疾病所致死亡风险是普通人群的2倍[4]。长随访研究显示，allo-HSCT后15年心血管事件发生率为7.5％[5]。allo-HSCT后5年冠心病发生率为3.2％～4.1％，心功能不全发生率为1.7％～3.0％[6]–[9]。与普通人群相比，移植患者心血管疾病发病年龄更低，如移植患者发生首次心肌梗死的中位年龄为53岁，而普通人群为67岁[10]。移植后冠心病发病与放疗（累及心脏区域）、高血压、糖耐量受损、血脂异常等有关[11]。移植后心功能不全发病的危险因素包括移植前化疗疗程（≥5个）、移植前蒽环类药物累积剂量、移植后严重急性GVHD、移植后重症感染等[6]–[7]。

（二）心血管危险因素（CVRF）

CVRF指与心血管疾病发生密切相关的代谢异常，主要包括高血压、糖尿病、血脂异常。allo-HSCT患者合并CVRF的风险也增加，移植后10年高血压、糖尿病、血脂异常的累积发病率分别为37.7％、18.1％、46.7％[4]。全身放射治疗（TBI）、糖皮质激素和钙调磷酸酶抑制剂的应用使CVRF的发病风险增加，而CVRF可促进移植后心血管疾病的发生。本共识结合国际骨髓移植登记组、欧洲骨髓移植登记组移植后心血管疾病筛查建议[11]和中国心血管病一级预防指南[12]作出以下推荐：

共识推荐：

1. 每年行心电图或24小时动态心电图检查，合并高危因素者可缩短复查间隔；若有新发症状或心电图变化，及时行冠脉CT或冠脉造影检查；

2. 移植前化疗疗程≥5个、移植后有严重合并症者每年行超声心动图检查监测心功能不全；无危险因素者每2～3年进行1次超声心动图检查；

3. 健康生活方式包括规律的锻炼计划和健康饮食计划，严格监测和管理血脂、血压和血糖水平（表1）。

二、非感染性肺部合并症

allo-HSCT后非感染性肺部合并症最常见的是闭塞性细支气管炎综合征（BOS）[13]。尽管最初通常轻微，但可逐渐进展至不可逆，显著增加移植后死亡率。BOS以非特异性炎症损伤影响小气道为特征，多发生在移植后2年内，也可在移植后4～5年发病。在所有allo-HSCT患者中BOS发生率为3％～6.5％[14]–[15]，在长期生存者中其发生率因生存时间延长和慢性GVHD的发生率增加而高达12％～14％[16]–[17]。BOS发生的最主要危险因素是进展型慢性GVHD，也与高龄、移植前气流受限、呼吸道病毒感染及预处理方案相关[14]–[18]。

BOS肺功能表现为阻塞性通气功能障碍，主要包括第一秒用力呼气容积（FEV1）下降（实测值低于预测值的80％）、FEV1与用力肺活量（FVC）的比值（FEV1/FVC％）显著下降（低于70％）[19]。高分辨率CT辅助诊断BOS应包括呼气相及吸气相扫描；主要影像表现为外周细支气管壁增厚、小气道扩张、“空气潴留征”和“马赛克衰减征”。病理学检查是诊断BOS的金标准，可表现为淋巴细胞性细支气管炎或缩窄性细支气管炎，但有创操作需谨慎[20]。BOS诊断分度及治疗原则参考《造血干细胞移植后闭塞性细支气管炎综合征诊断与治疗中国专家共识（2022年版）》[19]。

共识推荐：

1. 移植后2年内每3个月进行一次肺功能检测，有助于在免疫抑制剂减停期间筛查出患者，从而为早期干预提供机会；

2. 移植2年后每年行肺功能检查，若合并其他部位新发的慢性GVHD表现应缩短间隔；若有新发呼吸道症状（慢性咳嗽或呼吸困难），应及时复查肺功能检查并完善胸部CT；

3. 对于易发生肺部合并症的长期生存者，全身支持治疗包括感染预防（疫苗注射）、呼吸功能锻炼、严格戒烟、营养支持等。

三、内分泌系统疾病

（一）甲状腺疾病

甲状腺疾病包括亚临床和显性甲状腺功能减退。亚临床/代偿性甲状腺功能减退症的定义是促甲状腺激素（TSH）升高伴甲状腺素（T4）水平正常。相反，显性甲状腺功能减退的特征是TSH升高伴T4水平降低。亚临床甲状腺功能减退的发病率为25％～30％，中位发病时间为移植后2年；显性甲状腺功能减退的累积发病率为3.4％～9％，中位发病时间为移植后2.7年[21]。甲状腺功能减退与甲状腺区域放疗有关（累及颈/纵隔区域的放疗）。患者移植年龄越小，发病风险越高。

（二）骨骼系统

移植后骨骼系统疾病主要包括骨质疏松症和缺血性骨坏死，回顾性资料显示移植后长期生存者骨骼系统疾病的发生率为4％～40％[22]。移植后2年骨质疏松症发病率约为20％，骨质疏松症与应用糖皮质激素、生长激素水平降低、性腺功能减退、缺乏体育活动、维生素D缺乏和低钙饮食有关[10]。骨质疏松症患者常无症状，可通过双光能X线骨密度检查筛查发现；对于有症状（如骨痛）人群，需完善X线片明确是否合并非创伤性骨折。移植后缺血性骨坏死临床表现差异较大，大多数缺血性骨坏死发生在髋关节和膝关节，其危险因素包括移植时年龄较大、长期应用糖皮质激素、慢性GVHD、男性、肥胖等[10]。对于早期无症状人群，磁共振成像（MRI）检查可及时筛出。

**表1:** 造血干细胞移植后心血管危险因素（CVRF）的管理推荐

项目	筛查及管理
血脂	• 对于持续存在危险因素的患者(如应用钙调磷酸酶抑制剂、糖皮质激素)，建议每3~6个月评估一次；对于无上述持续危险因素的患者，年龄≥35岁的男性和年龄≥45岁的女性建议每年进行一次血脂评估
	• 若血脂异常，专科干预使低密度脂蛋白-胆固醇（LDL-C）<2.6 mmol/L
血压	• 推荐至少每6个月一次血压监测
	• 临界高血压（收缩压130~139 mmHg或舒张压85~89 mmHg）可先行非药物干预，包括适度限钠饮食，肥胖患者减轻体重和定期有氧运动
	• 高血压（收缩压≥140 mmHg或舒张压≥90 mmHg）者建议专科药物治疗，除非血压升高可归因于暂时性疾病或药物（如环孢素A）
血糖	• 对于存在持续危险因素的患者（如应用糖皮质激素），每3~6个月重复评估一次糖化血红蛋白（HbA1C）或空腹血糖；对于无上述持续风险患者，≥45岁成人每年筛查一次HbA1C或空腹血糖
	• 若明确血糖升高，建议采用生活方式治疗和专科药物治疗使HbA1C<7%

（三）性腺功能不全

移植患者生育能力下降和丧失主要由预处理方案的毒性所致，移植后性腺功能衰竭的发生率随着性腺毒性药物的累积剂量增加而增大，且预处理化疗常用药物中白消安较环磷酰胺对性腺功能影响更大[23]。此外，移植时年龄较大、女性患者、TBI为主的预处理方案均是生育能力丧失的危险因素。对于移植年龄<25岁且不合并慢性GVHD的男性患者，即使接受TBI为主的预处理方案，其生育能力仍有可能恢复。而卵巢功能更容易受到放疗和化疗的影响，育龄期女性患者接受清髓性化疗后早发性卵巢功能不全（POI）的发生率接近100％，受孕率<1％[24]。卵巢功能的不可逆性损伤使得移植后激素补充治疗（HRT）显得更为重要。对于育龄期女性，HRT的目的是缓解低雌激素的相关症状，对于无明显潮热等绝经症状的女性患者，也推荐HRT以维持骨骼、心血管系统、认知功能及泌尿生殖系统的健康，HRT启动时机建议待原发疾病情况稳定后再开始[25]。

对长期生存者而言，不能生育及由此带来的精神压力可能严重影响生活质量。因此，在移植前，医生与患者针对保留生育能力进行充分沟通是非常必要的。对于男性生育能力的保存，精子冻存技术已经比较成熟。对于女性患者，目前已应用于临床的卵巢功能保护措施包括胚胎冻存、成熟或未成熟卵母细胞冻存、卵巢组织冻存及药物暂时性抑制卵泡发育[26]。移植前使用促性腺激素释放激素激动剂（GnRHa）对卵巢的明确保护作用尚未得到证实[27]。

共识推荐：

1. 甲状腺：每年进行甲状腺功能的检查，显性甲状腺功能减退者应尽早行激素替代治疗。

2. 骨骼系统：

（1）每年行钙、磷、碱性磷酸酶、甲状旁腺激素和1,25-羟基维生素D水平测定及骨密度检查。

（2）无症状人群每3年进行下肢MRI（包括髋关节和膝关节）检查明确是否合并缺血性坏死；对于持续应用糖皮质激素者缩短至1.5年1次；若期间出现关节疼痛者，及时完善相应关节MRI。

（3）对于骨质疏松症且有症状者，及时进行药物干预；对于缺血性坏死患者，建议减轻负重、尽量减少糖皮质激素的应用、合并症状者及早专科干预。

3. 性腺：

（1）育龄期患者移植后每年进行性腺激素水平检测；

（2）移植后育龄期女性专科门诊评估激素替代治疗时机；

（3）生育保存能力：对于有生育需求的男性患者，可进行移植前精子冻存；对于有生育需求的女性患者，移植前专科咨询为其提供个体化的卵巢功能保护方案，具体包括胚胎冻存、成熟或未成熟卵母细胞冻存及卵巢组织冻存。

四、继发肿瘤

移植后另一个非常严重的合并症是二次肿瘤的发生[28]。宿主因素和临床特点均与移植后肿瘤发生风险密切相关，包括移植时年龄、移植前放化疗的暴露史、TBI为基础的预处理方案、致癌病毒的感染病史（EB病毒、乙型肝炎病毒、丙型肝炎病毒）及移植后免疫抑制治疗延长等[29]。本共识主要介绍继发实体瘤的发生和监测筛查。

allo-HSCT后15年实体瘤的累积发生率为7％～11％。移植患者实体瘤的中位发病年龄是55岁，而普通人群是67岁[30]。与年龄和性别匹配的普通人群相比，移植患者实体瘤发病的危险程度是其2倍多；在移植后随访15年或更长时间的患者中，实体瘤发病风险高达3倍。与实体瘤发生有关的因素包括：放疗（移植前和预处理TBI），致癌病毒感染（如乙型肝炎病毒、丙型肝炎病毒与肝癌，人乳头状瘤病毒与宫颈癌），慢性GVHD（如口腔慢性GVHD与鳞状细胞癌），吸烟者接受白消安为基础的预处理方案（肺癌）。此外，暴露于放疗时的年龄与实体瘤发生风险密切相关：30岁以下接受放疗的患者中，继发实体瘤发病风险是普通人群的9倍；而在30岁以上接受放疗的患者中，继发实体瘤发病风险接近普通人群。在移植后20年，皮肤基底细胞癌发病率约为6.5％，鳞状细胞癌发病率约为3.4％[28]。皮肤慢性GVHD与两者发病率增高均有关，而急性GVHD仅增加鳞状细胞癌的发病风险。在移植后25年，乳腺癌的发病率约为11％，是同龄健康人群的2.2倍，TBI可显著增加乳腺癌的发病率[30]。移植后甲状腺癌的中位发病时间是8.5年，移植患者甲状腺癌发病风险是年龄性别相匹配健康人群的3.3倍，颈部放疗、女性患者、慢性GVHD均增加甲状腺癌的发病风险[29]。腹部区域的放疗史、放疗剂量与结直肠癌的发病密切相关：与未接受放疗的人群相比，腹部区域接受10～29 Gy放疗患者胃肠肿瘤的发病率增加5.2倍，放疗剂量超过30 Gy的患者胃肠肿瘤发病率增加9.6倍[10]。

共识推荐：

1. 放疗区域皮肤软组织定期体格检查；

2. 甲状腺癌监测：每年行甲状腺功能及甲状腺彩超检查；

3. 乳腺癌监测：自青春期开始直到25岁每月进行乳房自检，每半年进行乳房体格检查；自25岁开始每年进行乳房彩超检查；

4. 早发型结直肠癌筛查：从35岁开始，每3～5年进行一次结肠镜检查。

五、移植后生活质量

关于移植后生活质量的研究报道并不少见。多数研究证实，移植后患者体能、社交、精神状态均稳步提高[31]–[32]，且在同胞全相合移植和单倍体移植中均呈逐年改善趋势[33]–[34]。慢性GVHD是影响移植后长期生活质量的重要因素。另外，工作能力是恢复正常生活的重要指标。在移植后2年，约75％的患者可重返工作岗位，未能返回工作岗位的最主要原因是活动性慢性GVHD所致健康状况不佳[10]。

关于心理健康，部分研究表明移植后第1年抑郁症发病率高达25％～35％，高于一般肿瘤人群的发病率[35]。与其他肿瘤患者群体相比，移植患者经历了更高强度的化疗、更大的经济压力以及更严重的并发症，都会造成更高水平的精神负担。在移植后长期生存者中，创伤后应激障碍（PTSD）发病率高达5％[36]。

共识推荐：

1. 移植后每年进行生活质量评分，成人应用SF-36量表或FACT-BMT量表，儿童应用PedsQL4.0量表。

2. 对于精神状态评估不佳者，应该考虑药物治疗或转诊到医学心理科室。

六、其他

慢性GVHD是比较常见的移植并发症，其发生率为30％～70％[37]，且慢性GVHD的出现是上述多种合并症的危险因素。慢性GVHD诊断及治疗建议参考《慢性移植物抗宿主病（cGVHD）诊断与治疗中国专家共识（2021年版）》[38]。此外，移植后免疫抑制剂应用、免疫重建延迟等因素均导致患者抵抗力下降，常合并移植后不同程度感染性疾病，需结合病原体、感染部位、患者免疫功能水平等综合评估诊疗。

综上，本共识聚焦移植后长期合并症管理计划，从心血管系统、呼吸系统、内分泌系统、继发实体瘤、生活质量评估等方面总结了不同系统长期合并症发生率、危险因素和筛查措施（表2）。在临床实践中应根据患者个体化暴露情况和风险因素实施长期预防和筛查，降低移植后长期生存者合并症的发生率及病死率，从而进一步提高移植后长期生存者生活质量。

**表2:** 造血干细胞移植后各系统合并症随访建议

组织/器官	对移植患者的随访建议
心血管系统	• 每年行心电图或24小时动态心电图检查；若有新发症状或心电图变化，及时行冠脉CT或冠脉造影检查
	• 高危人群每年行超声心动图检查监测心功能不全；无危险因素者每2～3年行超声心动图检查
	• 健康的生活方式，严格监测和管理血脂、血压和血糖水平
呼吸系统	• 移植后2年内每3个月进行一次肺功能检查
	• 移植2年后每年行肺功能检查，合并慢性GVHD者缩短间隔，若有新发呼吸道症状，及时复查肺功能检查并完善胸部CT检查
	• 全身支持治疗包括感染预防（疫苗注射）、呼吸功能锻炼、严格戒烟、营养支持等
骨骼肌肉系统	• 鼓励体育锻炼并补充维生素D和钙剂
	• 移植后每年进行钙、磷、碱性磷酸酶、甲状旁腺激素和1,25-羟基维生素D水平测定及骨密度检查
	• 无症状人群每3年进行下肢MRI检查；对于持续应用糖皮质激素者，检查间隔缩短至1.5年
生育力	• 移植前生育咨询（对于有生育需求的男性患者，可进行移植前精子冻存；对于有生育需求的女性患者，移植前专科咨询为其提供个体化的卵巢功能保护方案）
	• 移植后评估激素替代治疗时机（育龄期患者移植后每年进行性激素水平监测，移植后育龄期女性专科门诊评估激素替代治疗时机）
常见继发肿瘤	• 皮肤癌：放疗区域皮肤软组织定期的体格检查
	• 甲状腺癌监测：每年行甲状腺功能测定及甲状腺彩超检查
	• 乳腺癌监测：教育自青春期开始直到25岁每月进行乳房自检，每半年进行乳房体格检查；自25岁开始每年进行乳房彩超检查
	• 结直肠癌筛查：从35岁开始，每3～5年进行一次结肠镜检查
生活质量	• 每年进行生活质量量表评分
	• 对于精神状态评估不佳者，药物治疗或转诊到医学心理科室
